# The Impact of LDL Cholesterol, HDL Cholesterol, Triglycerides, and Vitamin D on Short-Term Implant Survival Rate: A Prospective Observational Study

**DOI:** 10.3390/jcm14103531

**Published:** 2025-05-18

**Authors:** Radu Ionut Grigoraș, Roberta Gasparro, Adina Simona Coșarcă, Timea Dakó, Alina Ormenișan

**Affiliations:** 1 IOSUD Doctoral School, George Emil Palade University of Medicine, Pharmacy, Science and Technology of Targu Mures, 38 Gheorghe Marinescu Street, 540142 Targu Mures, Romania; radu.grigoras@umfst.ro; 2 Department of Neuroscience, Reproductive Science and Dentistry, University of Naples Federico II, 80138 Naples, Italy; roberta.gasparro@unina.it; 3 Department of Oral and Maxillo-Facial Surgery, George Emil Palade University of Medicine, Pharmacy, Science and Technology of Targu Mures, 38 Gheorghe Marinescu Street, 540142 Targu Mures, Romania; alina.ormenisan@umfst.ro; 4 Department of Odontology and Oral Pathology, George Emil Palade University of Medicine, Pharmacy, Science and Technology of Targu Mures, 38 Gheorghe Marinescu Street, 540142 Targu Mures, Romania; timea.dako@umfst.ro

**Keywords:** cholesterol, triglycerides, vitamin D, dental implants, implant survival rate

## Abstract

**Background/Objectives**: Dental implant success is influenced by a range of systemic and local factors. Emerging evidence suggests that metabolic markers such as lipid profiles and vitamin D levels may play a role in osseointegration and implant survival. The aim of this study was to evaluate the influence of low-density lipoprotein (LDL) cholesterol, high-density lipoprotein (HDL) cholesterol, triglycerides, and vitamin D levels on the short-term survival rate of dental implants. **Materials and Methods**: A prospective observational study was conducted on patients receiving dental implants. Preoperative serum levels of LDL, HDL, triglycerides, and vitamin D were recorded. A total of 556 conical, platform-switching implants were placed in 166 patients, smokers and no smokers with mean age 48 years ± 4.7. Implant survival was evaluated from 14 to 21 days after placement, at 6- and at a 12-month follow-up. Spearman’s rank correlation was performed to assess potential correlations between the abovementioned systemic factors and implant loss. **Results:** Out of 556 implants, 13 (2.34%) were lost from 14 to 21 days after placement, a further two (0.35%) were lost after 6 months after surgery and a further eight (1.44%) were lost 12 months after placement. No significant correlation was found between HDL levels, cholesterol levels, triglyceride levels and implant loss. Spearman’s correlation analysis revealed a strong negative correlation between vitamin D levels and implant loss with no statistical significance. **Conclusions**: Within the limitations of this study, no statistically significant associations were found between lipid profile markers or vitamin D levels and early dental implant loss. Further large-scale and long-term studies are warranted to validate these findings and better understand the interplay between systemic biochemical markers and implant survival rate.

## 1. Introduction

Nowadays, implant therapy has become one of the most predictable methods for replacing one or more missing teeth, with a high long-term success rate [1,2].

Successful osseointegration is a fundamental requirement for implant therapy and is characterized by a functional ankylosis between the bone and the dental implant [3,4]. Osseointegration, defined as the direct structural and functional connection between living bone and the surface of a load-bearing artificial implant, is critical for the long-term success of dental implants [5]. The primary goal of any oral implantologist is to achieve optimal healing and ensure long-term treatment success. While implant design and surface innovations continue to evolve to have more reliable osseointegration, systemic health factors are often underappreciated [6]. The human skeletal system relies on essential minerals and vitamins such as calcium, fluoride, magnesium, potassium, vitamin B6, vitamin D, and zinc for proper function [7]. The European Registry of Nutrition and Health has identified 18 elements that directly influence bone and tooth health, including vitamin D [8], a micronutrient critical for implant osseointegration. Epidemiological studies estimate that vitamin D deficiency affects approximately 1 billion people globally, with older adults being particularly at risk—a demographic that also represents a significant proportion of dental implant patients [9]. Bone regeneration may be positively [10] or negatively [11] influenced by vitamin D levels. Deficiency in vitamin D has been linked to several conditions, including periodontitis [12,13], early tooth loss [14], catabolic metabolism, osteoporotic fractures [15], and delayed or impaired bone healing [16,17]. Vitamin D functions as a hormone synthesized in the skin through sun exposure and subsequently metabolized in the liver and kidneys [18]. However, in the northern hemisphere, most individuals do not receive sufficient sunlight to maintain adequate vitamin D levels. Alternative sources include fish oil, beef liver, eggs, milk, and mushrooms [19]. Vitamin D plays a critical role in bone metabolism, not only in bone formation but also in the regulation of alveolar bone resorption, which can lead to tooth loss. Animal studies have demonstrated that rodents with normal vitamin D levels exhibit active bone formation around dental implants, highlighting the direct relationship between vitamin D and osseointegration [20].

Emerging evidence suggests that serum lipid profiles, particularly levels of low-density lipoprotein (LDL) cholesterol, high-density lipoprotein (HDL) cholesterol, and triglycerides, may play a significant role in bone metabolism and inflammatory regulation [21]. Elevated LDL and triglyceride levels have been associated with increased systemic inflammation and oxidative stress, which could negatively impact bone healing and osseointegration [22]. In contrast, HDL cholesterol is believed to have anti-inflammatory and bone-protective properties [23]. These lipid-related mechanisms may influence the biological environment around dental implants, potentially affecting early healing, bone remodeling, and long-term implant stability [24].

In addition to systemic and biochemical factors, the local microbial environment significantly impacts the health and stability of peri-implant tissues. While peri-implantitis and periodontitis share certain clinical features, growing evidence highlights substantial differences in their microbiological profiles [25]. Unlike the more polymicrobial and diverse biofilms observed in periodontitis, peri-implant sites tend to harbor a less diverse but more pathogenic community, with higher proportions of anaerobic Gram-negative bacteria. This microbial shift contributes to a more aggressive inflammatory response in peri-implant tissues, which may explain the more rapid progression and higher incidence of bone loss seen in peri-implant lesions compared to periodontal ones. Understanding these microbial distinctions is critical when considering both implant prognosis and maintenance strategies, especially in patients with a history of periodontal disease [25].

Furthermore, bone mineral density (BMD), a local indicator of skeletal health, is known to affect implant anchorage and stability, particularly in cases where bone augmentation is not performed [26].

While some retrospective studies have examined these markers individually, there is a paucity of prospective clinical studies assessing the combined influence of lipid profiles and vitamin D levels on dental implant survival—especially in the early post-placement period, when biological stability is most vulnerable.

So, the aim of this prospective study was to evaluate the influence of low-density lipoprotein (LDL) cholesterol, high-density lipoprotein (HDL) cholesterol, triglycerides, and vitamin D levels on the short-term survival rate of dental implants.

## 2. Materials and Methods

### 2.1. Study Design and Setting

A prospective observational study was conducted on patients undergoing dental implant therapy at a private dental clinic, Dental D`OR, 53 Suceava Street, Târgu Mureș, Romania. All patients were thoroughly informed about the available treatment options and potential risks associated with implant therapy. Written informed consent was obtained from each participant prior to inclusion in the study. Data collection was performed using Microsoft Office Professional Plus 2019, version 2402.

This study was conducted in accordance with the ethical principles outlined in the Declaration of Helsinki and was approved by the Ethics Committee of the George Emil Palade University of Medicine, Pharmacy, Science, and Technology of Târgu Mureș, Romania (approval number 972/18 June 2020).

### 2.2. Patient Selection

For this study, 166 patients were included according to the following criteria:

1. Patients who needed at least one dental implant to be inserted;
2. Patients aged >18 years;
3. Patients without any systemic medical conditions that could contraindicate implant placement.

Exclusion criteria were as follows:

4. General conditions not compensated by medication;
5. General conditions that contraindicate implant treatment;
6. Conditions undergoing treatment with bisphosphonates;
7. Treatment with immunosuppressants;
8. Oncological treatment;
9. Pregnancy and lactation;
10. Bleeding and plaque index score > 25%.

### 2.3. Preoperative Evaluation

Relevant patient information was recorded, including age, sex, smoking habits, diabetes status, and other general health conditions. Preoperative serum levels of HDL, LDL, triglycerides, and vitamin D were measured 7 days prior to implant placement to ensure consistency and minimize biological variability.

An HDL value greater than 60 mg/dL was considered normal and indicative of low cardiovascular risk. Values between 40 and 59 mg/dL were classified as borderline risk, while HDL levels below 40 mg/dL were considered high risk for cardiovascular disease.

An LDL value greater than 190 mg/dL was considered indicative of significant cardiovascular risk. Levels between 150 and 190 mg/dL were classified as borderline risk, while LDL levels below 150 mg/dL were regarded as normal and associated with low cardiovascular risk.

A total cholesterol level greater than 240 mg/dL was considered indicative of significant cardiovascular risk. Levels between 200 and 240 mg/dL were classified as borderline risk, while values below 200 mg/dL were considered normal and associated with low cardiovascular risk.

Triglyceride levels greater than 200 mg/dL were considered indicative of significant cardiovascular risk. Levels between 150 and 200 mg/dL were classified as borderline risk, while levels below 150 mg/dL were regarded as normal and associated with low cardiovascular risk.

25-OH vitamin D levels were interpreted as follows: normal between 32 and 110 ng/mL, low between 20 and 32 ng/mL, and very low if below 20 ng/mL.

### 2.4. Implant Placement

OPT and CBCT scans were obtained for all patients to evaluate their eligibility for implant placement. Prior to implant placement, all patients received antibiotic prophylaxis. Surgeries were performed under local anesthesia (mepivacaine 1:100,000 adrenaline). After flap elevation, 556 conical platform-switching implants were placed in 166 patients. The implants were placed in both the maxilla and mandible, exclusively in native alveolar bone that did not require bone grafting. All implants achieved primary stability at the time of surgery, with insertion torque values exceeding 35–40 N/cm. All implants were left to heal without the use of a cover screw. After 6 months from implant placement, acrylic prosthesis was delivered. Nonsteroid analgesics were administrated after surgery. Sutures were removed 14 days postoperatively. All patients were recalled for maintenance hygiene program.

After local anesthesia, the oral mucosa incision was practiced in the edentulous area. In this way, a mucoperiostal flap with total thickness is created, exposing the alveolar bone. With the help of calibrated milling burs and respecting the milling sequence recommended by the manufactured, implant site is prepared. Insertion of the implants was performed respecting indications of the manufacturer. The flap was repositioned with simple or continuous sutures.

### 2.5. Follow-Up Period and Outcome Measures

The follow-up was considered from 14 to 21 days after surgery, 6 months after implant placement, and 12 months after placement (6 months after prosthetic loading). The 14–21-day interval was selected as the initial evaluation point to coincide with the early postoperative healing phase of peri-implant tissues. During this period, soft tissue healing is typically well underway, and early clinical signs of complications such as inflammation, mobility, or discomfort—potential indicators of implant failure—can be observed. At each time-point, the implant survival rate was recorded as the primary study outcome. Implant complications were the secondary outcome [27].

### 2.6. Statistical Analysis

Descriptive analysis of the study sample was achieved using IBM SPSS STATISTICS 25.0 for Windows™ software. Spearman’s rank correlation was performed to assess the relationship between LDL cholesterol, HDL cholesterol, triglycerides, vitamin D levels and implant loss.

## 3. Results

In total, 166 patients (118 females, 48 males; mean age: 48 years ± 4.7; age range 18–79 years) were enrolled in this study. Of the total number of patients included in the study, 128 (77.1%) were non-smokers and 38 (22.9%) were smokers. Patient characteristics at baseline are presented in Table 1.

Out of 556 implants, 13 (2.34%) were lost from 14 to 21 days after placement, a further two (0.35%) were lost after 6 months after surgery (total implants lost 15) and a further eight (1.44%) were lost 12 months after placement (total implants lost 23). No other implant complications were found.

The main findings on the implants’ survival rates according to serum levels of LDL cholesterol, HDL cholesterol, triglycerides, and vitamin D between 14 to 21 days postoperatively, at 6 months follow-up and at 12 months follow-up, are reported in Table 2, 3, and 4 respectively. Importantly, in Table 3 and Table 4, the number of implants lost refers to the additional losses observed at the specific follow-up time, relative to the number of implants placed. However, the calculation of the implant survival rate was based on the total number of implants lost, including those lost in previous follow-ups.

No significant correlation was found between HDL levels, cholesterol levels, triglyceride levels and implant loss. Spearman’s correlation analysis revealed a strong negative correlation between vitamin D levels and implant loss (Table 5).

## 4. Discussion

The aim of this study was to evaluate the influence of low-density lipoprotein (LDL) cholesterol, high-density lipoprotein (HDL) cholesterol, triglycerides, and vitamin D levels on the short-term survival rate of dental implants. Out of 556 implants, 13 (2.34%) were lost from 14 to 21 days after placement, a further two (0.3%) were lost after 6 months after surgery and a further eight (1.44%) were lost 12 months after placement.

Early implant failure typically occurs due to impaired osseointegration, often resulting from a lack of intimate contact between the bone and implant surface [28]. This can be attributed bacterial contamination, surgical trauma, or the use of blunt surgical instruments [29]. Additional contributors include inadequate bone density, lack of surgical guides leading to micromovements, and compromised healing [30]. Conversely, late implant failures are often associated with bruxism, a history of periodontitis, insufficient initial stability, or high bone density [31,32]. However, systemic factors such as abnormal lipid profiles and vitamin D deficiency have been proposed to negatively influence implant survival and osseointegration [33,34]. Despite this, relatively few studies have explored the relationship between serum lipid levels, vitamin D status, and dental implant outcomes [35].

Recent studies have highlighted a possible connection between hyperlipidemia and compromised bone health, with high-fat diets shown to reduce bone density and increase bone resorption [36,37]. Oxidized lipids have been implicated in the pathogenesis of osteoporosis, suggesting a link between lipid metabolism and skeletal integrity [38]. Hyperlipidemia has been shown to impact bone metabolism and potentially interfere with implant integration [39]. Understanding the molecular mechanisms underlying lipid interactions with bone could inform the design of future implant materials with dual osteogenic and lipid-modulatory functions. For patients with hyperlipidemia, preoperative management should include lipid profile normalization, and surgical techniques should aim to maximize primary stability and minimize trauma [40]. Despite this, our findings are consistent with several earlier studies, including those by Alsaadi et al., which reported no significant association between hypercholesterolemia and dental implant failure [41,42]. In our study, no meaningful correlations were observed between elevated levels of LDL, HDL, or triglycerides and implant loss. However, it is noteworthy that patients with normal HDL and triglyceride levels demonstrated a significantly higher rate of successful osseointegration, suggesting that a favorable lipid profile may support better implant outcomes, although further investigation is required to confirm this relationship.

No significant correlation was found between HDL levels, cholesterol levels, triglyceride levels and implant loss. The observed correlations are extremely weak, and statistical analysis does not provide evidence to support a link between HDL levels, cholesterol levels, triglyceride levels and implant loss. This discrepancy can be attributed primarily to the limited sample size, which reduces the statistical power to detect significant relationships even when the correlation coefficient is large. Additionally, variability in patient characteristics and confounding factors (e.g., systemic health status, bone quality, implant site) may have contributed to the wide confidence intervals and lack of significance. It is important to note that statistical non-significance does not necessarily imply the absence of a real effect. Further studies with larger cohorts are warranted to clarify these relationships.

Vitamin D plays a fundamental role in bone metabolism, immune regulation, and calcium-phosphate homeostasis [43,44]. Deficiencies in vitamin D have been associated with a variety of oral health issues, including periodontitis, reduced bone mineral density, delayed healing, and increased susceptibility to infections [45,46]. While some studies have reported improved implant outcomes following vitamin D supplementation, others have found no clear link between vitamin D receptor polymorphisms and implant survival [47]. In the present study, this correlation did not reach statistical significance, which aligns with existing literature indicating that the influence of vitamin D on implant osseointegration remains inconclusive [48]. Several studies have demonstrated that vitamin D supplementation may enhance osseointegration, particularly in systemic conditions such as osteoporosis, diabetes mellitus, or chronic kidney disease [49]. Moreover, vitamin D’s immunomodulatory properties and its ability to promote bone density homeostasis further justify its relevance in dental implant planning.

Given the accumulating evidence highlighting the importance of vitamin D in bone metabolism and osseointegration, evaluating and addressing vitamin D deficiency prior to dental implant placement appears both prudent and potentially beneficial. Accordingly, assessing each patient’s serum vitamin D status before implant surgery—and providing supplementation when warranted—may contribute to improved clinical outcomes [49,50]. However, this recommendation should be interpreted with caution, as conclusive validation through large-scale, randomized controlled trials in humans is still lacking [51,52], although patients with low serum vitamin D levels had a greater rate of early implant failure [53]. In addition to systemic supplementation, future research could explore the benefits of topical delivery systems for micronutrients such as vitamin D. Recent studies have highlighted the promising role of natural substances in oral care, including the use of toothpastes enriched with vitamin D as a preventive measure to support peri-implant tissue health and bone metabolism [54]. Integrating such approaches into daily hygiene routines could offer an accessible and patient-friendly strategy to enhance implant success, particularly for individuals with limited systemic absorption or chronic deficiencies.

In addition to systemic influences, postoperative factors such as oral hygiene play a pivotal role in the long-term success of dental implants [55]. Maintaining optimal oral hygiene after implant loading is essential for preventing peri-implant complications and ensuring implant longevity. In this regard, further research is warranted to better understand the impact of oral hygiene practices on implant outcomes and to develop evidence-based maintenance protocols.

Despite its findings, this study is subject to limitations, including a relatively small sample size and a short follow-up period. Additionally, the limited number of studies available on this specific topic constrains the ability to draw strong, generalizable conclusions. While Spearman’s correlation was used to assess associations between systemic markers and implant loss due to non-parametric distribution, multivariate analysis was not performed. As a result, the influence of potential confounding factors—such as age, smoking status, or bone density—could not be controlled for. This represents a limitation of the current study and will be addressed in future analyses.

Further large-scale, longitudinal studies are necessary to clarify the individual and combined effects of vitamin D status and lipid profiles on implant survival and bone metabolism. Expanding research in this area could lead to more personalized and effective treatment protocols for dental implant patients, particularly those with underlying systemic risk factors.

## 5. Conclusions

Within the limitations of this study, no statistically significant associations were found between lipid profile markers or vitamin D levels and early dental implant loss. Further large-scale and long-term studies are warranted to validate these findings and better understand the interplay between systemic biochemical markers and implant survival rate. In addition to traditional clinical protocols, future approaches should also consider proactive strategies, including both systemic and local interventions, aimed at enhancing peri-implant health. These may include stabilizing systemic biochemical parameters, such as LDL cholesterol and vitamin D, prior to implant therapy or daily use of adjunctive oral care products, such as toothpastes enriched with vitamin D, to support peri-implant bone and soft tissue health.

## Figures and Tables

**Table 1:** Patient characteristics at baseline.

Characteristics	Baseline
Total number of patients	166
Age (y), mean ± SD	48 ± 4.7
Age range (y)	18–79
Gender (male/female)	48/118
Total number of implants	556

**Table 2:** Implants lost/number of implants placed and implant survival rate according to serum levels of LDL cholesterol, HDL cholesterol, triglycerides, and vitamin D between 14 and 21 days.

	Normal Values	Borderline Values	Risk Values
LDL cholesterol	10/418 (97.6%)	0/58 (100%)	3/80 (96.2%)
HDL cholesterol	5/186 (97.3%)	8/334 (97.6%)	0/36 (100%)
Total cholesterol	5/280 (98.2%)	5/146 (96.6%)	3/130 (97.7%)
Triglyceride	4/384 (99%)	6/92 (94.5%)	3/80 (96.3%)
Vitamin D	1/54 (98.2%)	9/258 (96.5%)	3/244 (98.7%)

**Table 3:** Implants lost (n), number of implants placed, and implant survival rate according to serum levels of LDL cholesterol, HDL cholesterol, triglycerides, and vitamin D at 6-months follow-up.

	Normal Values	Borderline Values	Risk Values
LDL cholesterol	2/418 (0.47%)	0/58 (100%)	0/80 (100%)
HDL cholesterol	0/186 (100%)	2/334 (0.59%)	0/36 (100%)
Total cholesterol	0/280 (100%)	2/146 (1.37%)	0/130 (100%)
Triglyceride	0/384 (100%)	2/92 (2.17%)	0/80 (100%)
Vitamin D	0/54 (100%)	2/258 (0.77%)	0/244 (100%)

**Table 4:** Implants lost (n), number of implants placed, and implant survival rate according to serum levels of LDL cholesterol, HDL cholesterol, triglycerides, and vitamin D at 12-months follow-up.

	Normal Values	Borderline Values	Risk Values
LDL cholesterol	7/418 (67.2%)	0/58 (65.5%)	1/80 (65%)
HDL cholesterol	4/186 (69.3%)	4/334 (64%)	0/36 (78%)
Total cholesterol	4/280 (65.4%)	3/146 (68.5%)	1/130 (67.7%)
Triglyceride	4/384 (66.2%)	3/92 (68.5%)	1/80 (67.5%)
Vitamin D	1/54 (66.7%)	5/258 (65.9%)	2/244 (67.7%)

**Table 5:** Correlation between HDL, LDL, cholesterol, triglyceride, vitamin D levels and implant loss 12 months following implant placement.

Correlation	*p*-Value	r (Spearman’s Correlation)	CI
HDL vs. implant loss	0.1558	0.1107	−0.04691–0.2628
LDL vs. implant loss	0.4201	−0.06299	−0.2176–0.09470
Cholesterol level vs. implant loss	0.8883	−0.0109	−0.1674–0.1460
Triglyceride level vs. implant loss	0.1924	−0.1017	−0.05597–0.2544
Vitamin D vs. implant loss	0.2863	−0.0832	−0.07488–0.2369

CI: confidence interval.

## Data Availability

All data concerning this manuscript can be checked with the corresponding author at adina.cosarca@umfst.ro.
